# Hepatic Proteomic Analysis of Selenoprotein T Knockout Mice by TMT: Implications for the Role of Selenoprotein T in Glucose and Lipid Metabolism

**DOI:** 10.3390/ijms22168515

**Published:** 2021-08-07

**Authors:** Ke Li, Tiejun Feng, Leyan Liu, Hongmei Liu, Kaixun Huang, Jun Zhou

**Affiliations:** 1 Hubei Key Laboratory of Bioinorganic Chemistry & Materia Medica, School of Chemistry and Chemical Engineering, Huazhong University of Science and Technology, 1037 Luoyu Road, Wuhan 430074, China; like1992.hust@gmail.com (K.L.); andrewottertiejunfeng@gmail.com (T.F.); lly18337175085@gmail.com (L.L.); hongmeiliuhust@hust.edu.cn (H.L.); hxxzrf@hust.edu.cn (K.H.); 2 Shenzhen Huazhong University of Science and Technology Research Institute, Shenzhen 518057, China

**Keywords:** selenium, selenoprotein T, knockout, diabetes, glucose and lipid metabolism, proteomics, tandem mass tag

## Abstract

Selenoprotein T (SELENOT, SelT), a thioredoxin-like enzyme, exerts an essential oxidoreductase activity in the endoplasmic reticulum. However, its precise function remains unknown. To gain more understanding of SELENOT function, a conventional global *Selenot* knockout (KO) mouse model was constructed for the first time using the CRISPR/Cas9 technique. Deletion of SELENOT caused male sterility, reduced size/body weight, lower fed and/or fasting blood glucose levels and lower fasting serum insulin levels, and improved blood lipid profile. Tandem mass tag (TMT) proteomics analysis was conducted to explore the differentially expressed proteins (DEPs) in the liver of male mice, revealing 60 up-regulated and 94 down-regulated DEPs in KO mice. The proteomic results were validated by western blot of three selected DEPs. The elevated expression of Glycogen [starch] synthase, liver (Gys2) is consistent with the hypoglycemic phenotype in KO mice. Furthermore, the bioinformatics analysis showed that *Selenot*-KO-induced DEPs were mainly related to lipid metabolism, cancer, peroxisome proliferator-activated receptor (PPAR) signaling pathway, complement and coagulation cascades, and protein digestion and absorption. Overall, these findings provide a holistic perspective into SELENOT function and novel insights into the role of SELENOT in glucose and lipid metabolism, and thus, enhance our understanding of SELENOT function.

## 1. Introduction

Selenium (Se) is an essential trace element in humans. Dietary Se plays crucial roles in cancer prevention [1,2,3], aging [4], male reproduction [5], immune function [6] and other physiological and pathological processes [6]. Of particular biological significance, selenium exists in active sites of many selenoproteins in the form of selenocysteine [7]. Furthermore, selenoproteins are thought to be responsible for most of the biological functions of dietary selenium. Thus far, 24 kinds of selenoproteins in mice and 25 in humans have been identified [8].

Selenoprotein T (SELENOT, SelT) is one of seven endoplasmic reticulum (ER)-resident selenoproteins. It has a thioredoxin-like domain and possesses potent oxidoreductase activity [9], which strongly implies that SELENOT exerts an important redox activity that controls protein processing in the ER, allowing cells to respond to oxidative stress and to ensure ER homeostasis [10,11]. However, its precise function remains to be elucidated. Currently, constructing conventional and conditional selenoprotein knockout (KO) animal models has become an important tool for studying selenoprotein function. Interestingly, Boukhzar et al. reported that conventional whole animal Lox-Cre mediated deletion of exons 2 and 3 of *Selenot* is fetal lethal in mice [9], suggesting the indispensability of SELENOT during embryonic development. Of particular significance, Prevost et al. have reported that male conditional pancreatic β-cell *Selenot*-KO mice displayed impaired glucose tolerance and deficit in insulin production/secretion [12], indicating the involvement of SELENOT in glucose metabolism. However, how SELENOT affects glucose metabolism remains largely unknown, especially in insulin-responsive tissues such as the liver. To date, only one paper has reported the hepatic role of SELENOT, showing that SELENOT is induced during liver regeneration and exerts a cytoprotective effect [13].

With the development of molecular biology techniques, proteomic analysis has been widely applied in various studies [14]. Tandem mass tag (TMT) technology is a relative and absolute quantitative technology based on in vitro isotopic labeling introduced by Thermo Scientific, and commonly used in quantitative proteomics as a high-throughput screening technique [15]. This technique uses isotopic reagents to label amino groups at the end of polypeptides or amino groups at the side of lysine. By tandem analysis with high resolution mass spectrometer, the protein expressions of up to 16 samples can be compared at the same time.

To further explore the role of SELENOT in glucose metabolism, we firstly developed a conventional global *Selenot*-KO mouse model using a dual-small guide RNA (sgRNA) guided CRISPR/Cas9 strategy, and preliminarily analyzed its phenotype in the context of glucose metabolism. Given that the liver, one of the main insulin-responsive tissues, is central to glucose metabolism, in this study, we used TMT technology to analyze the protein composition changes in the liver between *Selenot*-KO and wild type (WT) mice, and further validated the changes of several important differentially expressed proteins (DEPs) using western blot.

## 2. Results

### 2.1. Generation and Metabolic Phenotype Analysis of Selenot KO Mice

**Figure 1:**
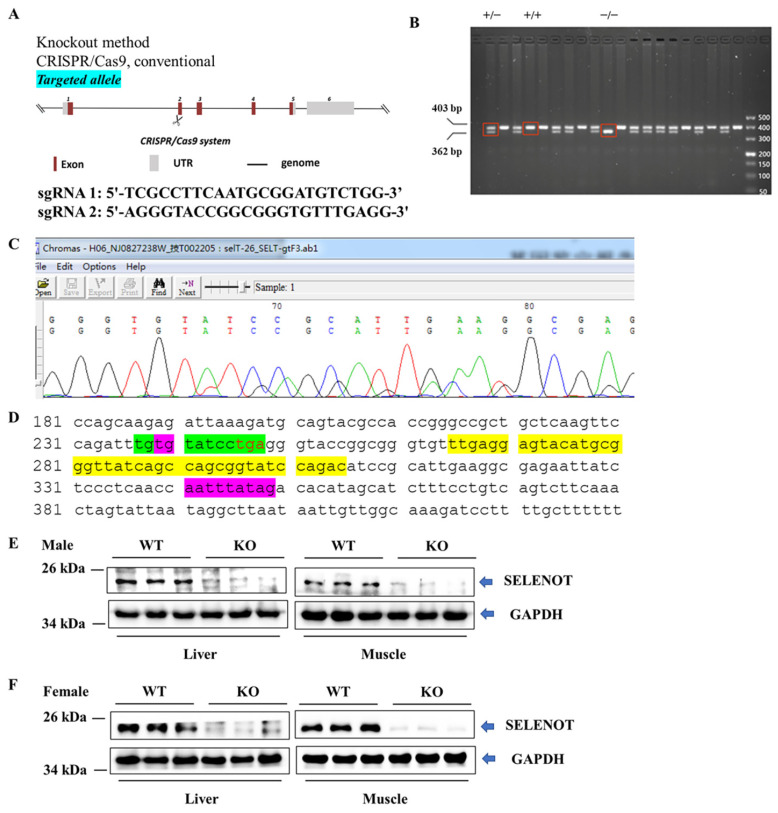
Establishment and identification of global *Selenot*-KO mice. (**A**) Global *Selenot*-KO mice were established via the CRISPR/Cas9 system, where dual sgRNA recognition sites were located on exon 2 of *Selenot*. (**B**) The agarose gel electrophoresis image of the PCR products of *Selenot* from the WT (+/+), heterozygote (+/−) and KO (−/−) mice. (**C**) Gene sequencing result for smaller bands in the PCR products of *Selenot* from heterozygous mice (Delete 41 bp on exon 2, resulting in frameshift GGTACCGGCGGGTGT-----------------------------------------ATCCGCATTGAA). (**D**) Partial gene sequence of *Selenot*. The gene sequence between the green highlighted parts corresponds to the active center of SELENOT, and the gene sequence between the purple highlighted parts is exon 2, and the yellow highlighted sequences are knocked out. (**E**) SELENOT protein content in liver and skeletal muscle from the male WT (+/+) and KO (−/−) mice detected by western blot (*n* = 3). (**F**) SELENOT protein content in liver and skeletal muscle from the female WT (+/+) and KO (−/−) mice detected by western blot (*n* = 6).

To ascertain if *Selenot* was knocked out, PCR was performed on mouse tail DNA with specific primers targeting exon 2 of *Selenot*. The PCR results showed the presence of normal (403 bp) and/or shift-mutated (362 bp) *Selenot* gene fragments (Figure 1B). Notably, in some samples only fragments of shift mutations were present, suggesting the successful generation of *Selenot*-KO (*Selenot^−/−^*) mice via the CRISPR/Cas9 system. Furthermore, the genetic sequencing result for smaller bands in the PCR products of *Selenot* from heterozygous mice showed that 41 bp was deleted in exon 2 of *Selenot*, resulting in frameshift GGTACCGGCGGGTGT-----------------------------------------ATCCGCATTGAA (Figure 1C). Meanwhile, genetic sequencing of homozygous mice yielded the same results. Moreover, the 41-bp deletion (yellow highlighted parts) did not include the gene sequence encoding the redox center of SELENOT, Cys-Val-Ser-Sec (green highlighted parts, Figure 1D). In addition, western blot was employed to detect protein expression of SELENOT in mice. The SELENOT protein was almost absent in the liver and skeletal muscle tissue from both male (Figure 1E) and female KO mice (Figure 1F), further confirming the success of *Selenot* knockout.

**Figure 2:**
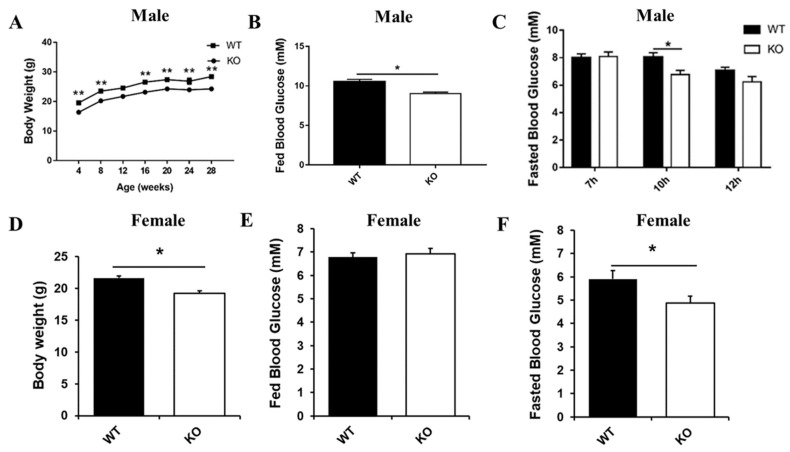
Metabolic phenotype analysis of *Selenot*-KO mice. Body weight (**A**) and fed blood glucose levels (**B**) of male KO and WT mice (*n* = 5). (**C**) The blood glucose levels of male KO and WT mice after fasting of different duration (*n* = 5). Body weight (**D**), fed blood glucose levels (**E**) and 10 h-fasting blood glucose levels (**F**) of female KO and WT mice (*n* = 6). Data were presented as means ± SEM. * *p* < 0.05, ** *p* < 0.01, compared with KO mice; ANOVA followed by a Mann–Whitney nonparametric *U* test.

Interestingly, our long-term observations showed that male *Selenot*-KO mice were infertile. Another noteworthy phenotype was that the *Selenot*-KO mice were obviously smaller in size than age-matched WT mice. In line with this, the body weight of both male and female *Selenot*-KO mice was significantly lower than sex- and age-matched WT mice (Figure 2A,D). To explore the effect of *Selenot*-KO on glucose metabolism, the blood glucose levels of the mice were analyzed. Compared with WT mice, the fed blood glucose levels (Figure 2B) and 10-h fasting blood glucose levels (Figure 2C) of male *Selenot*-KO mice decreased significantly. Moreover, although no significant changes in the fed blood glucose levels were observed (Figure 2E), female *Selenot*-KO mice displayed significantly decreased fasting blood glucose levels (Figure 2F), suggesting that SELENOT was involved in the control of glucose homeostasis. Moreover, male *Selenot*-KO mice displayed significantly decreased fasting serum insulin levels relative to WT mice (0.48 ± 0.05 ng/mL vs. 0.73 ± 0.07 ng/mL, *p* < 0.05, *n* = 5).

In addition, we detected some parameters for the serum lipid profile in male mice, including triglycerides (TG), total cholesterol (TC), HDL-C and LDL-C levels (Figure S1). Compared with male WT mice, TG, TC and LDL-C levels in the serum of male KO mice were significantly decreased, whereas there was no significant difference in serum HDL-C levels between the two groups. Overall, *Selenot* knockout could significantly improve the blood lipid profile in male mice.

### 2.2. Identification of Differentially Expressed Proteins (DEPs) by TMT Proteomic Analysis

**Figure 3:**
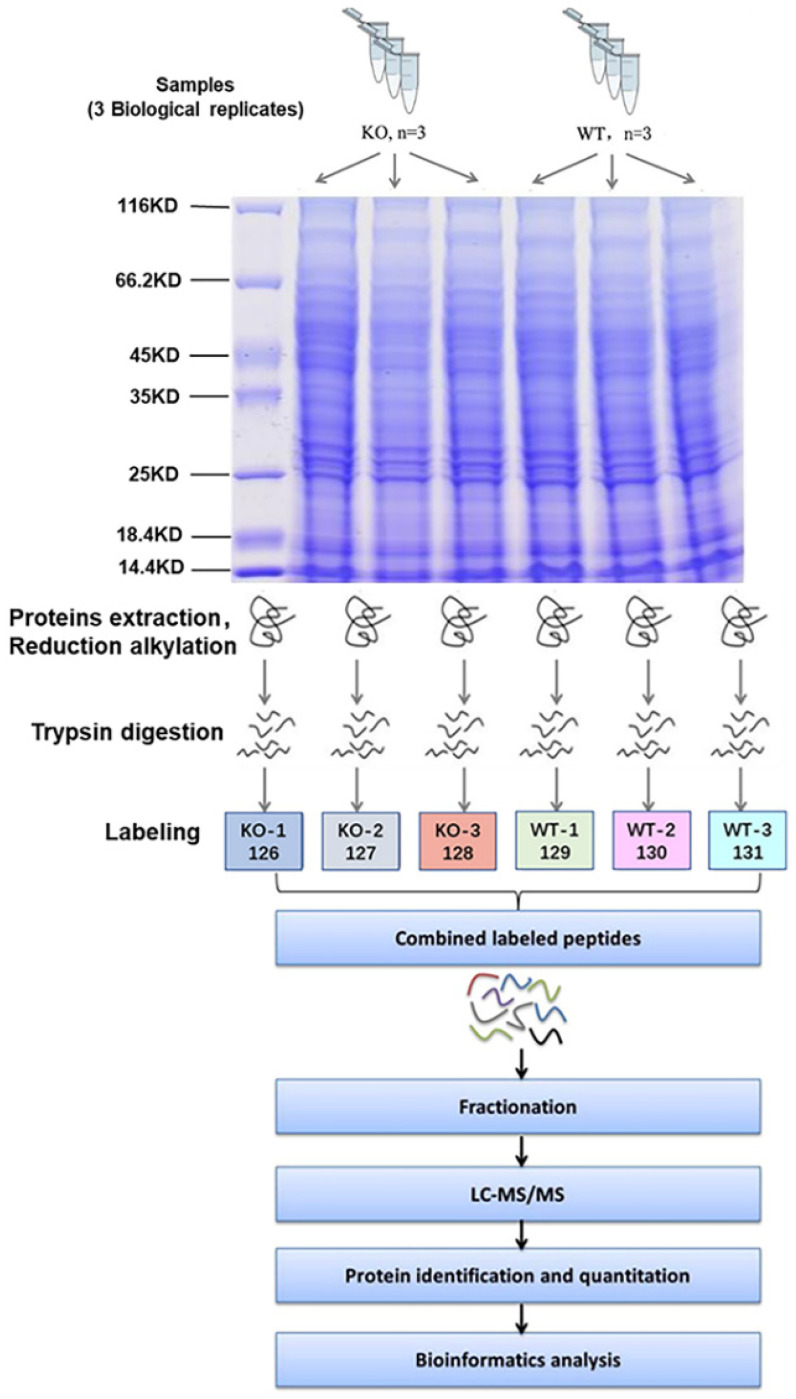
Flow chart of quantitative TMT proteomics experiments.

**Figure 4:**
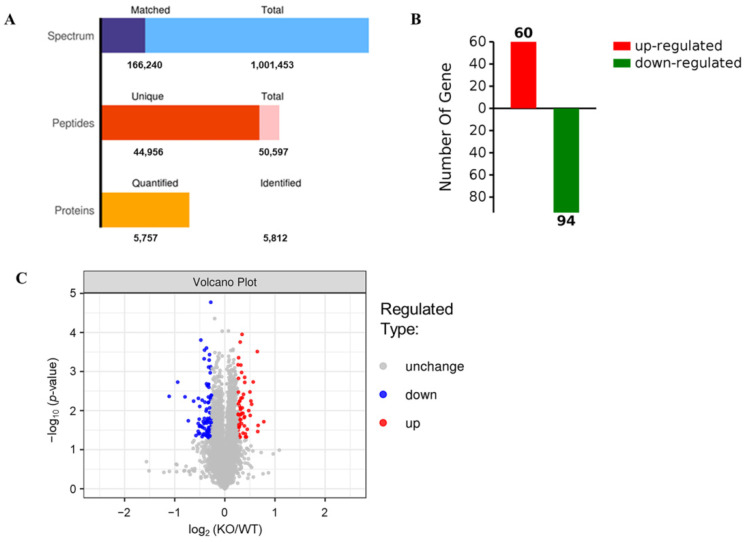
Differential expression of proteins detected by TMT in the livers of *Selenot*-KO and WT mice. (**A**) Numbers of spectrum, peptides and proteins. Total spectrum: the total number of secondary spectrograms; Matched spectrum: the total number of spectra matched the database. (**B**) Numbers of significantly up-regulated or down-regulated proteins in the livers of KO mice in comparison to WT mice. A protein was identified as significantly changed protein in the liver of KO mice if the FC was >1.2 (down < 0.83 times or up > 1.2 times), and the *p*-value was <0.05 compared with WT mice. (**C**) The volcano plots (log_2_ (KO/WT) vs. −log_10_ (*p*-value)).

The SDS-PAGE results showed that the protein bands from mouse livers were uniform and clear (Figure 3), suggesting that the samples did not degrade and could meet the quality control requirements for proteomic analysis. Therefore, a hepatic proteomic analysis was performed by TMT. As shown in Figure 4A, a total of 50,597 peptide fragments were identified through bioinformatics analysis, and 5757 quantifiable proteins were obtained. In order to analyze DEPs between the two groups, the experimental data were further screened for differences. After a statistical analysis, a protein was identified as significantly changed protein in the liver of KO mice if the fold change (FC) was > 1.2 (down < 0.83 times or up > 1.2 times), and the *p*-value was < 0.05 relative to WT mice. Based on the above criteria, a total of 154 DEPs were detected, including 94 down-regulated DEPs and 60 up-regulated DEPs (Figure 4B), and Table 1 and Table 2 give the specific information of the top 20 up-regulated and down-regulated proteins, respectively. The specific information of all DEPs can be found in Supplementary Table S1 (up-regulated DEPs) and Table S2 (down-regulated DEPs). Furthermore, the degree of difference in the DEPs between the two groups was also shown in the volcano plots (Figure 4C).

**Figure 5:**
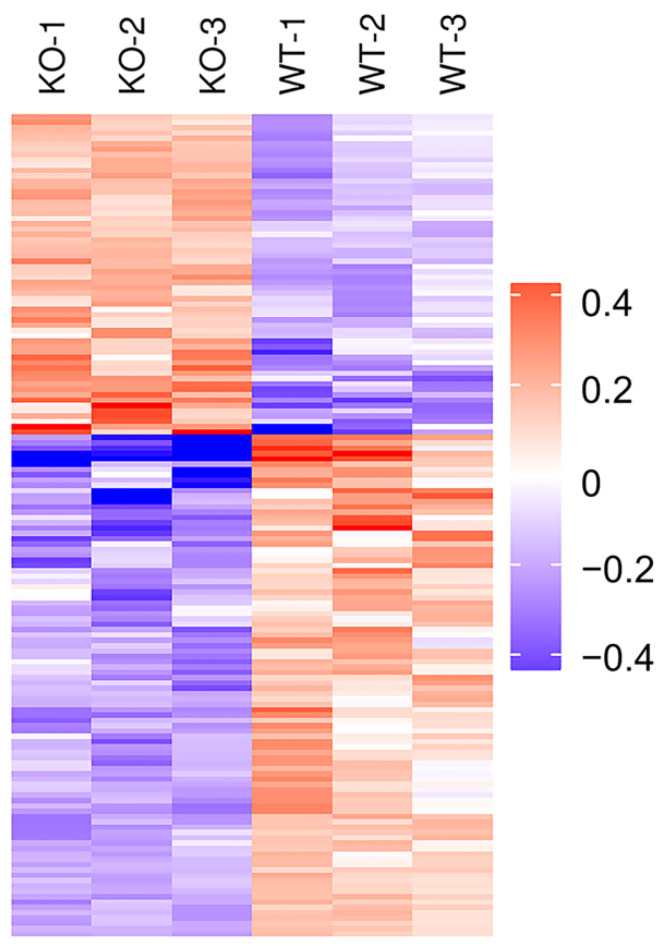
Cluster analysis of DEPs in *Selenot*-KO vs. WT group. In the heatmap, each column represents a sample (abscissa for sample information), and each row represents a protein (ordinate for DEPs). The expression levels of DEPs in different samples were standardized by Z-Score method and displayed in different colors. Among them, red represents significantly up-regulated proteins, blue represents significantly down-regulated proteins and gray represents proteins without quantitative information.

In order to analyze the expression patterns of samples between and within groups, to test the reasonableness of the grouping in this project and to show whether the changes in differential protein expression can represent the significant effects of biological treatment on the samples, the DEPs of the two groups were grouped and classified by Hierarchical Cluster and then displayed in the form of a heatmap. The clustering results showed that the similarity of data patterns within groups was high, while the similarity of data patterns between groups was low (Figure 5). Therefore, the DEPs obtained based on the above screening criteria can effectively distinguish the two groups, indicating that the DEPs screen can represent the impact of *Selenot*-KO on the samples.

### 2.3. Subcellular Localization Analysis of DEPs

**Figure 6:**
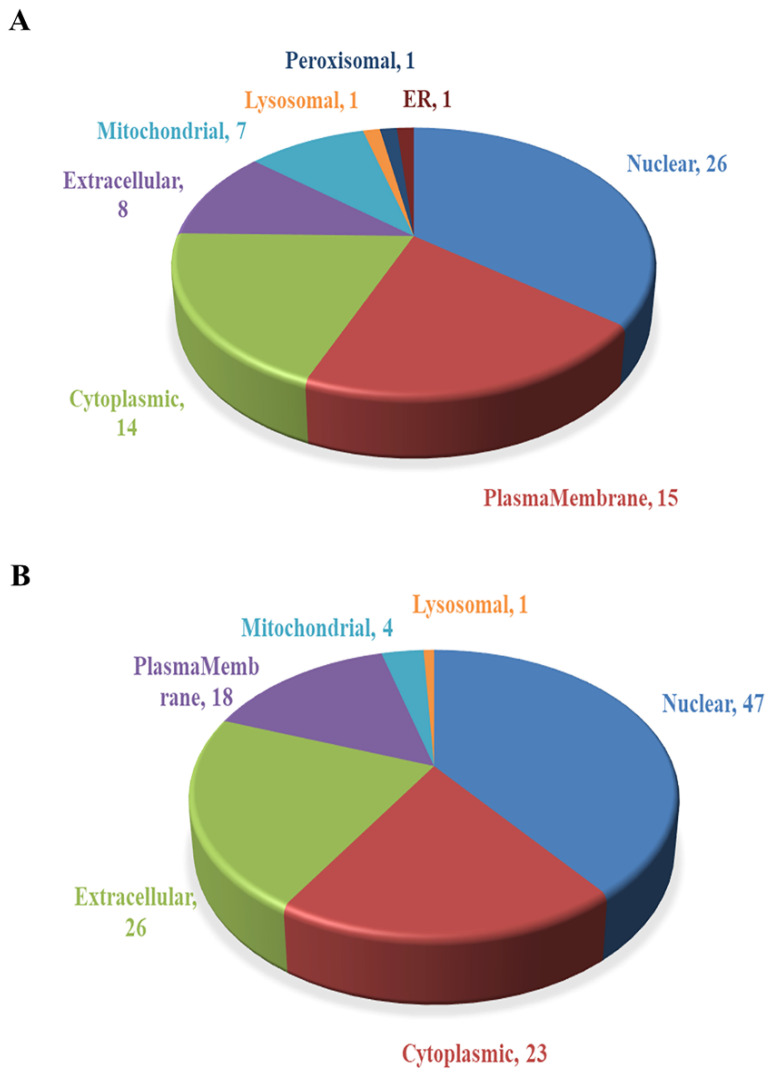
Subcellular localization of DEPs. (**A**) Subcellular localization of up-regulated DEPs. (**B**) Subcellular localization of down-regulated DEPs.

The subcellular localization prediction software CELLO was used to conduct subcellular localization analysis of all DEPs, showing the number and distribution ratio of DEPs in each subcellular organelle (Figure 6). The up-regulated DEPs are mainly located in the nucleus (26), plasma membrane (15) and cytoplasm (14). The down-regulated DEPs are mainly located in the nucleus (47), extracellular matrix (26), cytoplasm (23) and plasma membrane (18).

### 2.4. Domain Analysis of DEPs

**Figure 7:**
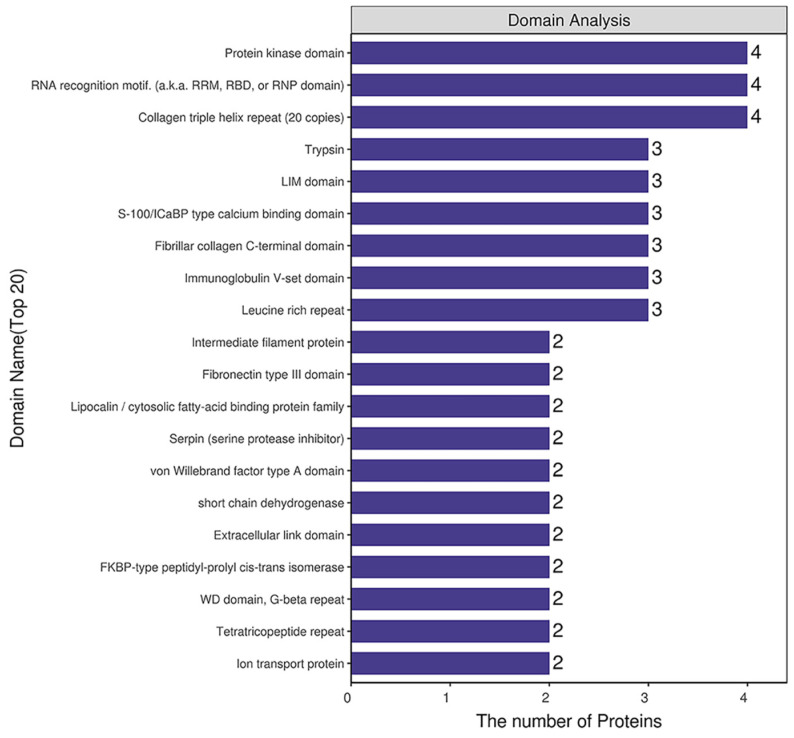
Structural domain analysis of DEPs.

Domain prediction software InterProScan was used to predict the domains of DEPs, and the number of proteins in the domains (top 20) was shown (Figure 7). The number of DEPs containing the protein kinase domain, RNA recognition motif (also knowns as the RRM, RBD or RNP domain) and the collagen triple helix repeat (20 copies) was the largest.

**Figure 8:**
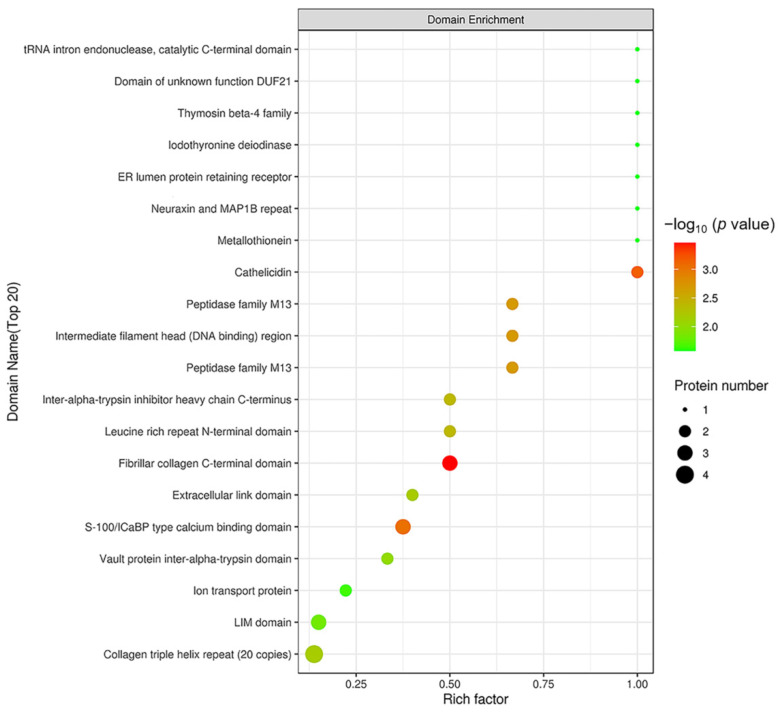
Structural domain enrichment analysis. The abscissa is the enrichment factor (Rich factor ≤ 1). The ordinate represents the statistical results of DEPs under each domain classification. The bubble color represents the significance of the enrichment of structural domain classification, namely, the *p* value was calculated using Fisher’s Exact Test and the color gradient represents −log_10_ (*p* value). The closer the color is to red, the smaller the *p* value and the higher the level of significance of the enrichment under the corresponding structural domain classification.

In order to reveal the structural domain enrichment characteristics of DEPs, and reveal significantly enriched domains and their corresponding DEPs by evaluating the significance level of protein enrichment in a certain domain, the domain enrichment analysis of DEPs was carried out using Fisher’s Exact Test. As shown in Figure 8, domains of the DEPs were mainly enriched in fibrillar collagen C-terminal domain, S 100/ICaBP type calcium binding domain and cathelicidin.

### 2.5. Gene Ontology (GO) Categorization of DEPs

**Figure 9:**
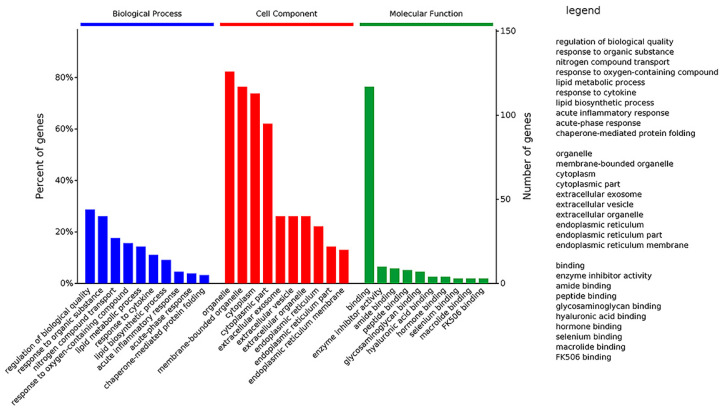
GO classification of DEPs. The DEPs were divided into three GO terms: Biological Process, Cell Component and Molecular Function.

For a comprehensive understanding of the function, localization and biological pathways of DEPs in living organisms, DEPs were annotated through Gene Ontology. GO functional annotations were mainly divided into three categories: Biological Process (BP), Cell Component (CC) and Molecular Function (MF) [16]. Figure 9 shows an overview of GO analysis with up to 10 significantly enriched terms in BP, CC and MF categories, respectively. The cut-off of *p*-value is set to 0.05.

GO analysis of DEPs showed that the top 10 significantly enriched terms in the Biological Process category are as follows: regulation of biological quality, response to an organic substance, nitrogen compound transport, response to an oxygen-containing compound, lipid metabolic process, response to a cytokine, lipid biosynthetic process, acute inflammatory response, acute-phase response, and chaperone-mediated protein folding. It implied that *Selenot*-KO is involved in regulation of biological quality and some responses related to inflammation and lipid metabolism.

Regarding the Cell Component, the top 10 significantly enriched terms are as follows: organelle, membrane-bounded organelle, cytoplasm, cytoplasmic part, extracellular exosome, extracellular vesicle, extracellular organelle, endoplasmic reticulum, endoplasmic reticulum part and endoplasmic reticulum membrane. These results showed that in the Cell Component category, most of *Selenot*-KO-induced DEPs are located in the (membrane-bounded) organelle in cytoplasm, suggesting that *Selenot*-KO might affect the function of some organelles, in particular the endoplasmic reticulum.

In the category of Molecular Function, binding was the most represented, in addition to enzyme inhibitor activity, amide binding, peptide binding, glycosaminoglycan binding, hyaluronic acid binding, hormone binding, selenium binding, macrolide binding and FK506 binding. It was very obvious that *Selenot*-KO-induced DEPs were mainly related to binding.

### 2.6. Analysis of Kyoto Encyclopedia of Genes and Genomes (KEGG) Pathways and Protein–Protein Interaction (PPI) among the DEPs

**Figure 10:**
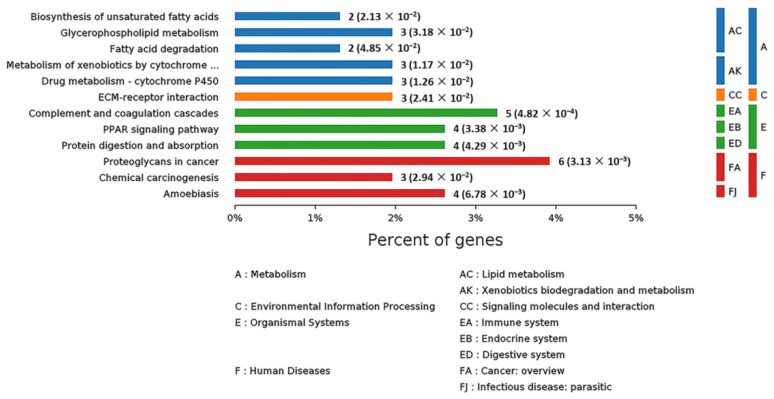
KEGG pathway analysis of the DEPs in livers of *Selenot*-KO and WT mice. According to the KEGG database, pathways are clustered into four sub-categories, (A) Metabolism, (C) Environmental Information Processing, (E) Organismal Systems and (F) Human Diseases.

In order to systematically and comprehensively analyze biological processes, disease mechanism, drug action mechanism, etc., it is often necessary to elucidate the law of changes from the perspective of a series of coordinated protein interactions, such as changes in metabolic pathways. Therefore, all the DEPs were subjected to KEGG pathway annotation through the KEGG pathway database [17]. At the same time, the number of proteins corresponding to DEPs was counted. All the statistically significant pathways were clustered into four sub-categories (Figure 10): Metabolism (13 proteins), Environmental Information Processing (1 protein), Organismal Systems (13 proteins) and Human Diseases (13 proteins). The specific information on all these proteins is listed in Table S3. As for human diseases, six, four and three proteins were related to proteoglycans in cancer, amoebiasis and chemical carcinogenesis, respectively. As for organismal information processing, five, four and four proteins were connected with complement and coagulation cascades, peroxisome proliferator-activated receptor (PPAR) signaling pathway and protein digestion and absorption, respectively. In the Metabolism sub-category, many DEPs were in connection with lipid metabolism, such as biosynthesis of unsaturated fatty acids, fatty acid degradation and glycerophospholipid metabolism. In consequence, SELENOT may play an important role in some diseases and in metabolism.

**Figure 11:**
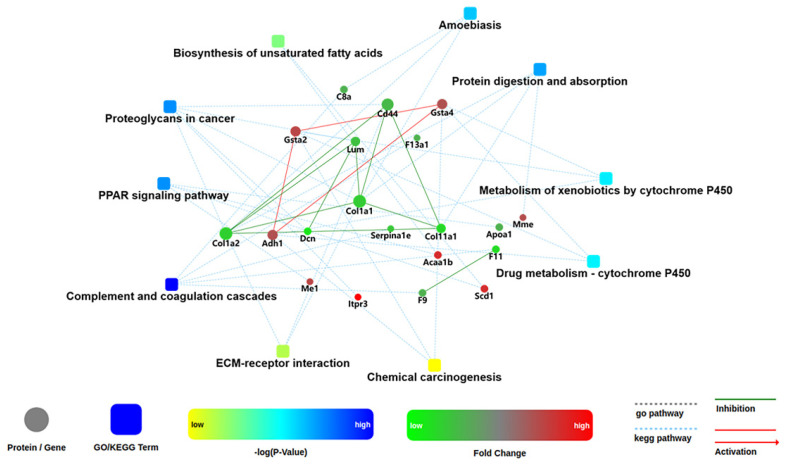
Network analysis of enriched pathways and interactions. Pathways were colored with a gradient color from yellow to blue; yellow for a smaller *p*-value, blue for bigger *p*-value. In case of fold change analysis, genes/proteins were colored in red (up-regulation) and green (down-regulation). The default confidence cutoff of 400 was used: interactions with bigger confident score were shown as solid lines between genes/proteins, otherwise in dashed lines.

One of the important ways for proteins to perform their functions is to interact with other proteins and play a biological regulatory role through interprotein-mediated pathways or complex formation. Therefore, PPI analysis is of great importance. The STRING (Search Tool for the Retrieval of Interacting Genes/Proteins) database was employed to retrieve the PPI information of the target proteins, and thereafter, the PPI network diagram for DEPs was generated with Cytoscape web application from OMICSBEAN, based on information gained up to four levels of functional analysis: fold change of gene/protein, biological process enrichment, KEGG pathway enrichment and protein–protein interaction. As shown in Figure 11, *Selenot*-KO-induced DEPs were mainly involved in complement and coagulation cascades, PPAR signaling pathway, some metabolic processes such as biosynthesis of unsaturated fatty acids, metabolism of xenobiotics by cytochrome, drug metabolism by cytochrome P450, proteoglycans in cancer, protein digestion and absorption and ameobiasis.

### 2.7. Validation of DEPs by Western Blotting

**Figure 12:**
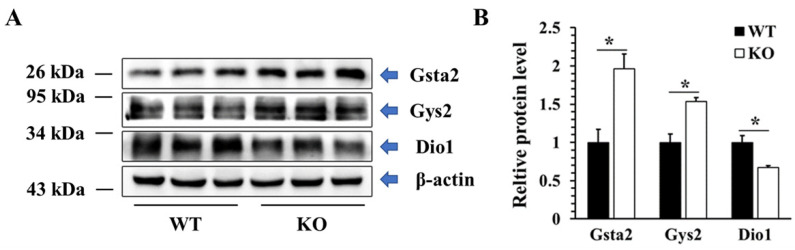
Validation of DEPs at the protein level. Representative western blot (**A**) and quantified data (**B**) of selected DEPs in WT and KO mice. Data were presented as the fold of the WT group and as means ± SEM (*n* = 3). * *p* < 0.05; ANOVA followed by a Mann–Whitney nonparametric *U* test.

To validate the proteomic data, western blot technology was used to detect the protein levels of three DEPs in different KEGG pathways, including two up-regulated proteins (Gsta2, Gys2) and one down-regulated protein (DIO1). As shown in Figure 12, the protein levels of Gsta2 and Gys2 were increased by 96% and 53%, respectively, while the protein level of DIO1 was decreased by 33%, which were consistent with the results of proteomics.

## 3. Discussion

As one of the seven ER selenoproteins, SELENOT is highly expressed in mouse and human β-cells and other endocrine tissues and is involved in intracellular Ca^2+^ mobilization and neuroendocrine secretion [12,13,18,19]. Moreover, SELENOT contains a thioredoxin-like domain and possesses potent oxidoreductase activity as a thioredoxin reductase (TXNRD, TrxR)-like enzyme [9]. Recently, there has been increasing interest in exploring the biological function of SELENOT. It has been reported that SELENOT could protect dopaminergic neurons in mouse models of Parkinson’s disease because of its crucial oxidoreductase activity [9]. In a recent study, *Selenot* knockdown aggravated cisplatin-induced apoptosis in NRK-52E cells, a kind of rat renal tubular epithelial cells, indicating that SELENOT prevented mice from cisplatin-induced acute kidney injury (AKI) by suppressing oxidative stress and cell apoptosis [20]. However, the function of SELENOT remains little known. Currently, genetically engineered animal models are an important means of studying the effects of certain genes or proteins on organisms and life forms, and thus, they have been widely used. In this regard, glutathione peroxidase 1 (GPX1)^−/−^ mice [21], the 15-kDa selenoprotein (SELENOF, Sep15) KO mice [22,23], selenoprotein P (SELENOP, SEPP1) KO mice [24] and some other selenoprotein KO mice [25] have been constructed successfully and used in related researches. Notably, Boukhzar et al. tried to construct conventional *Selenot*-KO mice but failed, because they showed that global *Selenot*-KO led to death during the embryonic period [9]. Consequently, this group has constructed several conditional *Selenot*-KO mouse models, including conditional pancreatic β-cell *Selenot*-KO mice [12] and conditional brain *Selenot*-KO mice [9], advancing research on the roles of SELENOT in neuroprotection [9,26] and glucose metabolism [12]. Intriguingly, male conditional pancreatic β-cell *Selenot*-KO mice displayed impaired glucose tolerance and a deficit in insulin production/secretion [12], suggesting that SELENOT is involved in glucose metabolism by disrupting insulin production/secretion. However, whether SELENOT can regulate glucose metabolism in insulin-responsive tissues remains unknown, mainly due to the lack of corresponding genetically engineered animal models. In the present study, we have successfully constructed a conventional global *Selenot*-KO (*Selenot*^−/−^) mouse model using a CRISPR/Cas9 strategy, as evidenced by genotyping and western blotting. We deleted 41 bp in exon 2 of *Selenot*, resulting in shift-mutated *Selenot* gene fragments. Surprisingly, this global *Selenot*-KO mouse model is survivable, contrary to the results reported by Boukhzar et al. [9]. This discrepancy may come from the difference in the deletion region of *Selenot*. Boukhzar et al. deleted exons 2–3 of *Selenot*, which contain the putative redox center of SELENOT, Cys-Val-Ser-Sec [9]. It has been reported that SELENOT is abundant in embryonic hearts but undetectable in adult hearts, which suggested SELENOT played an important role in the development of the embryonic heart [27]. Moreover, in ischemia/reperfusion injury model, a SELENOT-derived peptide encompassing the redox motif, which is key to its function, conferred cardioprotection through inhibition of oxidative stress and apoptosis [27]. In contrast, a control peptide lacking the redox site failed to protect heart. Accordingly, complete deletion of exons 2 (compassing the redox site) and 3 might cause severe impairment or loss of SELENOT function, thus inducing fetal lethality. In contrast, in the current study the deletion of 41 bp did not include the redox center of SELENOT, which resulted in retention of part of SELENOT function in *Selenot*-KO mice, thus making mouse survival possible. Notably, although the male *Selenot*-KO mice are infertile, the heterozygous (*Selenot*^+/−^) mice are fertile and can be used for breeding. Interestingly, the genotype ratios of homozygous, heterozygous and WT mice in litters are 14:55:31, suggesting this KO also has reduced embryonic survival. It is notable that the hydrophobic amino acid sequences at positions 87–102 and 125–143 of SELENOT may represent transmembrane domains and may be required for anchoring SELENOT to ER [19,28]. In line with this, modeling studies suggest that these hydrophobic segments contain amphipathic helices that interface with the ER membrane allowing partial binding and insertion of SELENOT [29]. In our *Selenot*-KO mouse model, although the redox center of SELENOT is retained, these hydrophobic amino acid sequences of SELENOT are deleted, possibly hindering its ER localization and, thus, partially compromising its function. This hypothesis is supported by the fact that our *Selenot*-KO mice are partially fetal lethal, similar to the global *Selenot*-KO mice reported by Bukhzar et al. Therefore, the *Selenot*-KO model presented in this paper may not be a very ideal model, but it still provides an optional tool for studying the function and structure–function relationship of SELENOT. To our knowledge, this is the first conventional global *Selenot*-KO mouse model.

It is well recognized that selenium deficiency would cause male sterility. Given the fact that knockout of mitochondrial glutathione peroxidase 4 (mGPx4) causes complete loss of male fertility of mice [30], mGPx4 is the only selenoprotein known to play a critical role in male fertility to date. Notably, in adult rats, the expression levels of SELENOT are low in most tissues, but it remains particularly abundant in endocrine organs, such as pancreas, thyroid and testis [13]. Furthermore, in the testis, SELENOT is found in the testosterone-producing Leydig cells and also the proliferating and differentiating spermatogenic cells. However, to date the role of SELENOT in male fertility remains unknown. Based on our findings, it is possible that deletion of SELENOT may affect spermatogenesis and, thus, cause sterility in mice. Therefore, our findings suggest SELENOT as another selenoprotein that is critical for male fertility. Nevertheless, further investigations are warranted to elucidate the role of SELENOT in male fertility and the underlying mechanisms.

Next, we observed some differences in mouse phenotypes between WT and *Selenot*-KO mice during the study period. Of particular significance, *Selenot*-KO mice displayed reduced size and body weight relative to age-matched WT mice. To explore the role of SELENOT in glucose metabolism, the blood glucose levels of the mice were further detected. Surprisingly, *Selenot*-KO led to significantly lower fed and/or fasting blood glucose levels. This phenotype is opposite to the phenotype of conditional pancreatic β-cell *Selenot*-KO mice, which displayed higher blood glucose levels relative to WT mice following glucose loading, despite normal fasting glucose levels [12]. Mechanistically, the impaired glucose tolerance in the conditional pancreatic β-cell *Selenot*-KO mice was attributed to the reduction in glucose-stimulated insulin secretion [12]. In the current study, given the lower fasting serum insulin levels in the male *Selenot*-KO mice, it is reasonable to speculate that their hypoglycemic phenotype may be attributed to the promotion of insulin sensitivity. Therefore, it is evident that *Selenot*-KO in tissues other than the pancreas affects glucose metabolism in a different way from pancreatic β-cell *Selenot*-KO, especially in insulin-responsive tissues. In addition, *Selenot*-KO in male mice also led to significantly lower serum TG, TC and LDL-C levels, suggesting the potential of SELENOT in improving the blood lipid profile. Overall, these findings imply a novel and important relationship between SELENOT and glucose and lipid metabolism.

To obtain more information about SELENOT function, we next used TMT technology to analyze *Selenot*-KO-induced DEPs in the liver, a main insulin-responsive tissue. A total of 5757 proteins were identified, including 60 up-regulated DEPs and 94 down-regulated DEPs. For a comprehensive understanding of the function, localization and biological pathways of these DEPs in living organisms, DEPs were annotated through GO analysis. Based on the analysis data, most of the DEPs are located in nucleus, and many DEPs are located in cytoplasm, extracellular matrix as well as plasma membrane. The cytoplasm is the main site of metabolism. ER is a membrane-bound organelle which is involved in protein synthesis, processing and transport, lipid synthesis and calcium homeostasis [31]. In terms of the biological process, a lot of DEPs take part in the metabolic process, such as Gys2. Gys2 is one of two isoforms of glycogen synthase [32], and also a rapid-limiting enzyme catalyzing insulin-mediated liver glycogen synthesis. Several studies have shown that the activation of Akt in the insulin-mediated pathway can lead to phosphorylation of glycogen synthase kinase 3 (GSK3), thereby reducing its inhibition of Gys2 and promoting glycogen synthesis [33,34]. At the same time, insulin can also directly enhance the activity of Gys2 and promote the synthesis of glycogen [35]. Our data have shown that hepatic Gys2 was significantly elevated in *Selenot*-KO mice, which would promote the entry of more blood glucose into hepatocytes for glycogen synthesis, consistent with the lower blood glucose levels observed in *Selenot*-KO mice. Therefore, it is reasonable to speculate that SELENOT KO may lower blood glucose levels via modulating the expression of some metabolism-related proteins in the liver, such as Gys2.

Many DEPs are involved in biological regulation, including Dio1. The major biological function of Dio1 is to catalyze the conversion of thyroxine (T4) to triiodothyronine (T3) [36]. Dio1 plays a role in the generation of plasma T3 by deiodination of T4 in peripheral tissues such as the liver and kidney, and thus, provides most of the circulating T3 essential for growth, differentiation and basal metabolism in vertebrates. Virtually any serious disease is related to a decrease in T3 in circulation, a condition known as the nonthyroidal illness syndrome (NTIS) [37]. Interestingly, our data showed that Dio1, as a selenoprotein, was also significantly decreased in the liver of *Selenot*-KO mice. The reduction in Dio1 expression may result in low circulating T3 levels, impeding the growth of mice and eventually leading to reduced size and body weight in the *Selenot*-KO mice. Some studies have shown that selenium deficiency would lead to a decrease in expression of Dio1, which depends on the organs being studied, because some organs can be resistant to the lack of selenium in diet [38,39,40], even though Dio1 was observed to reduce in some diseases. In this regard, a 50–60% decrease in hepatic Dio1 was observed in experimental diabetes in rats [36]. Moreover, Dio1 activity has been altered in some tumor types [41]. Altogether, these data suggest an association between a reduction in Dio1 and some diseases. However, the mechanisms and consequences of the reduction in Dio1 caused by *Selenot*-KO are poorly understood, and further follow-up studies are needed.

In order to have a comprehensive understanding of the biological process, disease mechanism, etc., KEGG analysis was performed. Based on the data, some DEPs were found to be involved in human diseases, including proteoglycans in cancer and chemical carcinogenesis, which are related to cancer. Specifically, the up-regulated DEPs in the *Selenot*-KO mice include Itpr3, Gsta2, Adh1 and Gsta4, while the down-regulated DEPs include Cd44, Lum, Col1a1, Col1a2 and Dcn. In this regard, a variety of selenoproteins have been demonstrated to influence the development of cancer in different directions: global SELENOP haploinsufficiency augments tumorigenesis and mediates oxidative damage in the intestine [42]; Thioredoxin reductase 1 plays a role in cancer promotion [43,44,45]; SELENOF (Sep15) plays a stimulatory role in colon cancer [46,47]. However, whether and how SELENOT acts in cancer remain unknown to date. Since oxidative stress is one of the principal characteristics of cancer cells [43], SELENOT is predicted to influence cancer development. The current proteomic results support this hypothesis and provide the first evidence for the relationship between SELENOT and cancer. Moreover, the KEGG result has also revealed that *Selenot*-KO is related to the immune system. In line with this, an experiment performed in Caenorhabditis elegans confirmed that SELENOT was required for avoidance of the bacterial pathogens *Pseudomonas aeruginosa* and *Serratia marcescens* [48]. Overall, the KEGG analysis of DEPs has provided new directions for research into the role of SELENOT in human diseases and organismal systems, including but not limited to the role of SELENOT in lipid metabolism-related diseases, cancer and complement system deficiency diseases.

Another noteworthy discovery was a significant up-regulation of protein level of hepatic Gsta2 in *Selenot*-KO mice. Gsta2 is a cis-regulatory element or enhancer sequence, which is found in the promoter region of several genes encoding detoxification enzymes and cytoprotective proteins [49]. Recent studies suggest that the up-regulation of Gsta2 may be a compensatory mechanism against elevated oxidative stress [50,51]. In our mouse model, *Selenot*-KO may reduce the body’s antioxidant capacity, thereby leading to a compensatory increase in the expression of other antioxidant enzymes, such as Gsta2.

Some DEPs are involved in the lipid metabolism and PPAR signaling pathway. Specifically, in terms of biosynthesis of unsaturated fatty acids, Acaa1b and Scd1 were up-regulated in the *Selenot*-KO mice; in terms of fatty acid degradation, Acaa1b and Adh1 were up-regulated in the *Selenot*-KO mice; in terms of glycerophospholipid metabolism, Cept1 and Pemt were up-regulated, while Gpcpd1 was down-regulated in the *Selenot*-KO mice; in terms of PPAR signaling pathway, Acaa1b, Scd1 and Me1 were up-regulated, while Apoa1 was down-regulated in the *Selenot*-KO mice. Lipid homeostasis is regulated by a complex network of interconnected signaling pathways, among which the PPAR signaling pathway plays a pivotal role in lipid production [52]. Our data suggest that *Selenot*-KO may regulate the expression of lipid metabolism-related proteins, such as PPAR signaling molecules, to control lipid homeostasis. Given the interplay between glucose and lipid metabolism, further study of these DEPs will help to elucidate the phenotypes of *Selenot*-KO mice observed in this study, including reduced blood glucose levels and body weight. On the other hand, based on these findings, it is necessary to investigate the phenotype of lipid metabolism and its mechanisms in *Selenot*-KO mice in the following study.

Despite its nutritional benefits, selenium overexposure has been positively associated with type 2 diabetes (T2D) and high-grade prostate cancer according to recent randomized trials [53]. Moreover, several recent meta-analyses of both nonexperimental and experimental studies indicate that moderate to high levels of selenium exposure are associated with increased risk for T2D [54,55]. In this context, the study focusing on the relationship between selenoproteins and diabetes has received considerable attention, indicating unsuspected key roles of SELENOP and GPX1 in diabetes [56,57]. In this regard, SELENOP levels are elevated in T2D patients, and excessive SELENOP causes insulin resistance and also impairs the function of pancreatic β-cells, suggesting excessive SELENOP as a promising therapeutic target in T2D patients [58,59,60,61]. Our results suggest that SELENOT KO may have the potential to ameliorate disorders of glucose and lipid metabolism, raising the possibility that excess SELENOT may be another selenoprotein involved in the pro-diabetic effects of high selenium intake, although the molecular mechanisms remain unclear. In addition, given that the role of SELENOT in diabetes seems to be somewhat similar to that of SELENOP, whether and how SELENOT and SELENOP interact with each other is an interesting topic. Nevertheless, further investigation is warranted to elucidate these issues.

## 4. Materials and Methods

### 4.1. Animals

Animals were housed in a temperature (22 ± 2 °C)-controlled room with a 12-h day/night cycle, and supplied with adequate standard laboratory chow (0.20 ± 0.02 mg Se/kg diet) and water unless otherwise noted. Animal procedures were approved by the Institutional Laboratory Animal Ethics Committee at Huazhong University of Science and Technology (Wuhan, China; s1900, approval date: 3 June 2019), and were conducted according to the institutional guidelines.

The global *Selenot*-KO C57BL/6J mice were generated by Nanjing Biomedical Research Institute of Nanjing University (Nanjing, Jiangsu, China) using the CRISPR/Cas9 technique. Briefly, dual sgRNA (5′-TCGCCTTCAATGCGGATGTCTGG-3′ and 5′-AGGGTACCGGCGGGTGTTTGAGG-3′) directed Cas9 endonuclease cleavage of the *Selenot* gene and created a double-strand break that would be repaired and lead to frameshift from exon 2 (Figure 1A). The targeted genomes of F0 mice were amplified by PCR and sequenced and the chimeras were crossed with WT C57BL/6J mice to obtain F1 mice. Since male homozygous *Selenot*-KO (*Selenot*^−/−^) mice were sterile, heterozygous mice (*Selenot*^+/−^) were used for reproduction. Furthermore, the offspring mice were genotyped by tail clipping, DNA isolation, PCR (*Selenot* forward primer: 5′-TGGGGCATAAACATACAAAG-3′, reverse primer: 5′-AAGAACCAGCAAGGGTGACT-3′) and gel electrophoresis.

According to the genotyping results, homozygous mice (KO) with similar birth dates were finally chosen for follow-up experiments. WT age-matched C57BL/6J mice were selected as the control group, and thereafter, the phenotypes of mice in the two groups were observed. The mice were weighed weekly, and the blood glucose levels of mice were detected by an ACCU-CHEK Active glucometer (Roche, Mannheim, Germany). At the end of the experiment, the mice (11-month-old) were anesthetized with chloral hydrate, blood was taken from the orbit and then the mice were sacrificed and dissected. The pancreas, liver, adipose tissue, kidney and other tissues of the mice were removed and stored in the −80 °C refrigerator until analysis. The SELENOT protein was determined by western blotting from mouse tissues, including liver and skeletal muscle.

### 4.2. Proteomic Analysis

A TMT-based quantitative proteomic approach was employed to analyze the proteome in the liver. The whole process of proteomics analysis mainly includes two stages: mass spectrometry experiment and data analysis. The process of mass spectrometry analysis mainly includes extraction of proteins, enzymatic hydrolysis of peptides, TMT labeled chromatography, LC-MS/MS data acquisition and database retrieval (Figure 2).

#### 4.2.1. Protein Extraction and Digestion

Three male *Selenot*-KO mice and three male WT mice (7–8 months old) were chosen for the proteomic analysis. SDT (4% SDS, 1 mM DTT, 100 mM Tris-HCl, pH 7.6) buffer was employed to lyse the liver tissue and extract proteins. The samples were centrifuged for 15 min at 12,000× *g* (4 °C), and then the BCA Protein Assay Kit (Bio-Rad, Hercules, CA, USA) was used to quantify the protein concentrations of the supernatant. For protein quality control, a qualitative analysis of protein samples was performed using SDS-PAGE prior to proteomic studies, and the protein bands were visualized by Coomassie Blue staining.

Proteins were digested with trypsin according to a filter-aided sample preparation (FASP) procedure [62]. Briefly, 200 μg of proteins for each sample were added into 30 μL SDT buffer (150 mM Tris-HCl, 100 mM DTT, 4% SDS, pH 8.0) for reduction. After repeated ultrafiltration (Microcon units, 10 kD), 100 mM iodoacetamide (IAA) was added to block reduced cysteine residues, followed by an incubation for 30 min in darkness. After multiple washing, the protein suspensions were digested overnight with 4 μg trypsin (Promega, Madison, WI, USA) in NH_4_HCO_3_ buffer (40 μL, 25 mM) at 37 °C. Finally, the digested peptides were desalted on C18 Cartridges (Empore™ SPE Cartridges C18, Sigma, St. Louis, MO, USA), concentrated by vacuum centrifugation and reconstituted in 0.1% (*v*/*v*) formic acid.

#### 4.2.2. TMT Labeling

TMTsixplex™ reagent was employed to label the peptide mixture (100 μg) of each sample according to the manufacturer’s instructions (Thermo Fisher Scientific, Waltham, MA, USA). Briefly, TMT reagent was thawed, reconstituted in acetonitrile and then mixed with peptide sample. The peptide mixtures were incubated for 1 h at room temperature and pooled, desalted and dried by vacuum centrifugation.

#### 4.2.3. High pH Reversed-Phase Fractionation

Labeled peptides were fractionated by High pH Reversed-Phase Peptide Fractionation Kit according to the manufacturer’s instructions (Thermo Fisher Scientific). The dried peptide mixture was dissolved and acidified with 0.1% trifluoroacetic acid solution and loaded to the equilibrated, high-pH, reversed-phase fractionation spin column. After desalting peptides with water, a step gradient of increasing acetonitrile concentrations in a volatile high-pH elution solution was applied to the columns to elute bound peptides, which were then merged into 18 different fractions. These fractions were desalted on C18 Cartridges and then concentrated by vacuum centrifugation.

#### 4.2.4. LC-MS/MS Analysis

LC-MS/MS analysis was performed on a Q Exactive mass spectrometer (Thermo Fisher Scientific) that was coupled to EASY nLC 1000 UPLC system (Proxeon Biosystems, now Thermo Fisher Scientific) for 60/90 min. The peptides were dissolved in 0.1% formic acid aqueous solution and loaded onto a reversed-phase trap column (Thermo Fisher Scientific Acclaim PepMap100), and then separated by a C18 reversed-phase analytical column (Thermo Fisher Scientific EASY column, C18-A2). Mobile phase A was 0.1% formic acid aqueous solution and mobile phase B was 84% acetonitrile and 0.1% formic acid aqueous solution. The column was equilibrated with 95% mobile phase A, and the peptides were separated with a linear gradient of buffer B at a flow rate of 300 nL/min. The mass spectrometer was operated in positive ion mode. The scanning range of parent ions was 300–1800 *m/z*, the resolution of primary mass spectrometry was 70,000 at 200 *m/z*, the AGC (Automatic Gain Control) target was 1e6, the maximum inject time (IT) was 50 ms and the Dynamic Exclusion time was 30.0 s. The mass and charge ratios of peptides and peptide fragments were collected according to the following methods: after each full scan, 20 fragments (MS2 Scan) were collected; the MS2 activation type was HCD, the isolation width was 2 *m/z*, the secondary mass spectral resolution was 17,500 at 200 *m/z*, the Normalized Collision Energy was 30 eV and the underfill ratio was defined as 0.1%. The instrument was run with the peptide recognition mode enabled.

#### 4.2.5. Identification and Quantitation of Proteins

The raw MS data for each sample were RAW files, and the software Mascot 2.2 and Proteome Discoverer 1.4 were employed for library identification and quantitative analysis. Relevant parameters and explanations are as follows: Enzyme was set as Trypsin; Max Missed Cleavages were 2; Peptide Mass Tolerance was ±20 ppm; Fragment Mass Tolerance was 0.1 Da; Fixed modifications were carbamidomethyl (C) and TMT 6plex (N-term and K), and variable modifications were methionine oxidation and TMT 6plex (Y); Database was Swissprot_mouse_17042_20200217.fasta; Database pattern for calculating FDR (false discovery rate) was Decoy; Peptide and protein FDR was ≤0.01. As for protein quantification, the protein ratios were calculated as the median of only unique peptides of the protein. As for experimental bias, all peptide ratios were normalized by the median protein ratio. The proteomics data are openly available in ProteomeXchange with identifier PXD023261.

### 4.3. Bioinformatics Analysis

#### 4.3.1. Protein Cluster Analysis

Firstly, the quantitative information of the target protein set was normalized to the interval (−1, 1). Next, the ComplexHeatmap R package (R Version 3.4, Zuguang Gu, German Cancer Research Center, Heidelberg, Germany) was used to categorize the sample and protein expression in two dimensions (Euclidean distance algorithm and Average linkage clustering algorithm), and the hierarchical clustering heatmap was generated.

#### 4.3.2. Subcellular Location Analysis

CELLO (http://cello.life.nctu.edu.tw/, accessed date: 22 July 2020), which uses multi-class support vector machine (SVM)-based machine learning methods to model protein sequence data with known subcellular localization information in public databases, was used to predict the subcellular localization information of the proteins to be retrieved [63].

#### 4.3.3. Protein Structure Domain Analysis

The protein domains were analyzed using the Pfam database. The InterProScan software package was used to run the scan algorithm from the InterPro database in an integrated manner to perform the functional characterization of sequences, thus obtaining the domain annotation information of the target protein sequences in the Pfam database [64].

#### 4.3.4. GO Analysis, KEGG Pathway Analysis and PPI Network Analysis

The GO categorization, KEGG pathway enrichment and PPI network analysis were performed by uploading the data from the TMT experiments to OMICSBEAN (http://www.omicsbean.cn/, accessed date: 17 September 2020), and then the online software would produce results.

### 4.4. Western Blot

The liver or skeletal muscle tissue was homogenized using RIPA lysis buffer (Beyotime, Shanghai, China) containing 1% protease inhibitor cocktail (Merck, Darmstadt, Germany), and then centrifuged for 15 min at 12,000× *g* (4 °C). The protein content of the supernatant was quantified with a BCA kit (Applygen, Beijing, China). The protein samples were subjected to SDS-PAGE, and thereafter transferred to PVDF membranes (Millipore, Billerica, MA, USA). After blocking with 1% BSA TBS-T solution, the membranes were incubated with primary antibodies against GAPDH (Beyotime, AG019-1, 1:1000), SELENOT (Abcam, ab176192, 1:1000), Type I iodothyronine deiodinase (DIO1; Santa Cruz, Dallas, TX, USA, sc-515198, 1:100), Glutathione S-transferase A2 (Gsta2; Thermo Fisher Scientific, PA5-100255, 1:500) and Glycogen [starch] synthase, liver (Gys2; Santa Cruz, sc-390391, 1:100), respectively, and subsequently secondary antibodies conjugated with horseradish peroxidase (Biosharp, Hefei, Anhui, China, BL001A/BL003A). Finally, the membranes were visualized with ECL kit (Millipore, Billerica, MA, USA) using a Tanon 5200 Automatic Chemiluminescence Imaging Analysis System (Tanon, Shanghai, China).

### 4.5. Statistical Analysis

Statistical analysis was performed by ANOVA followed by a Mann–Whitney nonparametric *U* test. A value of *p* < 0.05 was considered statistically significant.

## 5. Conclusions

In this study, conventional global *Selenot*-KO (*Selenot*^−/−^) mice were successfully constructed for the first time using the CRISPR/Cas9 technique. The *Selenot*-KO mice exhibited male sterility, reduced size/body weight, lower fed and/or fasting blood glucose levels and lower fasting serum insulin levels and improved blood lipid profile, which imply a novel and important relationship between SELENOT and glucose and lipid metabolism. TMT proteomics analysis showed 154 differentially expressed proteins in the liver of male *Selenot*-KO mice, including 60 up-regulated and 94 down-regulated proteins. The elevated Gys2 expression is consistent with the hypoglycemic phenotype in KO mice. Furthermore, *Selenot*-KO-induced DEPs were mainly related to lipid metabolism, cancer, PPAR signaling pathway, complement and coagulation cascades and protein digestion and absorption, suggesting an association between SELENOT and disorders of glucose and lipid metabolism as well as cancer. Overall, these findings provide a holistic perspective into SELENOT function and novel insights into the role of SELENOT in glucose and lipid metabolism, and suggest new directions for research into the role of SELENOT in human diseases.

## Figures and Tables

**Table 1:** Up-regulated hepatic DEPs in *Selenot*-KO mice (top 20).

Uniprot ID	Gene Name	Fold Change KO/WT	Protein Name	*p* Value
P70227	Itpr3	1.7142	Inositol 1,4,5-trisphosphate receptor type 3	0.0193
Q810C0	Slitrk2	1.5838	SLIT and NTRK-like protein 2	0.0240
Q99JH8	Kdelr1	1.5758	ER lumen protein-retaining receptor 1	0.0344
P19426	Nelfe	1.5667	Negative elongation factor E	0.0003
Q80V94	Ap4e1	1.4803	AP-4 complex subunit epsilon-1	0.0019
Q8C4X7	Minar2	1.4486	Major intrinsically disordered NOTCH2-binding receptor 1-like homolog	0.0069
Q8VCH0	Acaa1b	1.4334	3-ketoacyl-CoA thiolase B, peroxisomal	0.0057
Q80X71	Tmem106b	1.4200	Transmembrane protein 106B	0.0133
P13516	Scd1	1.4148	Acyl-CoA desaturase 1	0.0033
Q9CR30	Josd2	1.3915	Josephin-2	0.0100
Q6P1E7	Primpol	1.3635	DNA-directed primase/polymerase protein	0.0301
Q9EQQ2	Yipf5	1.3481	Protein YIPF5	0.0472
Q80ZJ8	Cracr2b	1.3299	EF-hand calcium-binding domain-containing protein 4A	0.0473
E9Q2M9	Wdfy4	1.3271	WD repeat- and FYVE domain-containing protein 4	0.0395
Q9JIA7	Sphk2	1.3167	Sphingosine kinase 2	0.0019
Q8BGS7	Cept1	1.3154	Choline/ethanolaminephosphotransferase 1	0.0014
Q9JMI0	Ecel1	1.3130	Endothelin-converting enzyme-like 1	0.0143
Q6P2L6	Nsd3	1.3102	Histone-lysine *N*-methyltransferase NSD3	0.0247
Q02242	Pdcd1	1.3066	Programmed cell death protein 1	0.0151
Q920M5	Coro6	1.2939	Coronin-6	0.0038

**Table 2:** Down-regulated hepatic DEPs in *Selenot*-KO mice (top 20).

Uniprot ID	Gene Name	Fold Change KO/WT	Protein Name	*p* Value
Q61646	Hp	0.7401	Haptoglobin	0.0418
Q71KU9	Fgl1	0.7342	Fibrinogen-like protein 1	0.0267
Q01149	Col1a2	0.7313	Collagen alpha-2(I) chain	0.0054
P11588	Mup1	0.7303	Major urinary protein 1	0.0464
A2AAE1	Kiaa1109	0.7279	Uncharacterized protein KIAA1109	0.0259
C0HKD8	Mfap1a	0.7176	Microfibrillar-associated protein 1A	0.0002
Q8BL00	Cdhr3	0.7118	Cadherin-related family member 3	0.0243
Q1ERP8	Cd300lg	0.7068	CMRF35-like molecule 9	0.0384
Q61245	Col11a1	0.7067	Collagen alpha-1(XI) chain	0.0078
Q91Y47	F11	0.7040	Coagulation factor XI	0.0253
Q91WR5	Akr1c21	0.7019	Aldo-keto reductase family 1 member C21	0.0166
P97315	Csrp1	0.6954	Cysteine and glycine-rich protein 1	0.0391
Q9D304	Rnf128	0.6938	E3 ubiquitin-protein ligase RNF128	0.0049
P09602	Hmgn2	0.6922	Non-histone chromosomal protein HMG-17	0.0214
P28654	Dcn	0.6919	Decorin	0.0339
Q9QY24	Zbp1	0.6706	Z-DNA-binding protein 1	0.0435
P14602	Hspb1	0.6493	Heat shock protein beta-1	0.0057
A2ASS6	Ttn	0.6033	Titin	0.0182
Q63836	Selenbp2	0.5763	Selenium-binding protein 2	0.0045
P02802	Mt1	0.5208	Metallothionein-1	0.0019

## Data Availability

The proteomics data presented in this study are openly available in ProteomeXchange with identifier PXD023261.

## Supplementary Materials

The following are available online at https://www.mdpi.com/article/10.3390/ijms22168515/s1, Table S1: Up-regulated hepatic DEPs in *Selenot*-KO mice; Table S2: Down-regulated hepatic DEPs in *Selenot*-KO mice; Table S3: Statistically significant pathways in KEGG pathway analysis of the DEPs in livers of *Selenot*-KO and WT mice; Figure S1: Serum TG, TC, HDL-C, LDL-C levels in male WT and *Selenot*-KO mice.
