# Supplementary material for: Hepatic Proteomic Analysis of Selenoprotein T Knockout Mice by TMT: Implications for the Role of Selenoprotein T in Glucose and Lipid Metabolism
Source: Int J Mol Sci. 2021 Aug 7;22(16):8515. doi: 10.3390/ijms22168515 (PMC8395235; doi:10.3390/ijms22168515)
Supplement: Supplementary file 1 [file ijms-22-08515-s001.zip › ijms-1316197-supplementary.pdf]

Table S1. Up-regulated hepatic DEPs in *Selenot*-KO mice

Uniprot ID	Gene Name	Fold Change KO/WT	Protein Name	p value
P70227	Itpr3	1.7142	Inositol 1,4,5-trisphosphate receptor type 3	0.0193
Q810C0	Slitrk2	1.5838	SLIT and NTRK-like protein 2	0.0240
Q99JH8	Kdelr1	1.5758	ER lumen protein-retaining receptor 1	0.0344
P19426	Nelfe	1.5667	Negative elongation factor E	0.0003
Q80V94	Ap4e1	1.4803	AP-4 complex subunit epsilon-1	0.0019
Q8C4X7	Minar2	1.4486	Major intrinsically disordered NOTCH2-binding receptor 1-like homolog	0.0069
Q8VCH0	Acaa1b	1.4334	3-ketoacyl-CoA thiolase B, peroxisomal	0.0057
Q80X71	Tmem106b	1.4200	Transmembrane protein 106B	0.0133
P13516	Scd1	1.4148	Acyl-CoA desaturase 1	0.0033
Q9CR30	Josd2	1.3915	Josephin-2	0.0100
Q6P1E7	Primpol	1.3635	DNA-directed primase/polymerase protein	0.0301
Q9EQQ2	Yipf5	1.3481	Protein YIPF5	0.0472
Q80ZJ8	Cracr2b	1.3299	EF-hand calcium-binding domain-containing protein 4A	0.0473
E9Q2M9	Wdfy4	1.3271	WD repeat- and FYVE domain-containing protein 4	0.0395
Q9JIA7	Sphk2	1.3167	Sphingosine kinase 2	0.0019
Q8BGS7	Cept1	1.3154	Choline/ethanolaminephosphotransferase 1	0.0014

Q9JMI0	Ecel1	1.3130	Endothelin-converting enzyme-like 1	0.0143
Q6P2L6	Nsd3	1.3102	Histone-lysine N-methyltransferase NSD3	0.0247
Q02242	Pdcd1	1.3066	Programmed cell death protein 1	0.0151
Q920M5	Coro6	1.2939	Coronin-6	0.0038
Q5SS00	Zdbf2	1.2855	DBF4-type zinc finger-containing protein 2 homolog	0.0377
Q9D413	Sh2d6	1.2842	SH2 domain-containing protein 6	0.0117
P10648	Gsta2	1.2728	Glutathione S-transferase A2	0.0047
P06801	Me1	1.2721	NADP-dependent malic enzyme	0.0085
O54909	Rdh16	1.2688	Retinol dehydrogenase 16	0.0001
Q5SWP3	Nacad	1.2621	NAC-alpha domain-containing protein 1	0.0117
Q61907	Pemt	1.2610	Phosphatidylethanolamine N-methyltransferase	0.0011
Q8VCB3	Gys2	1.2594	Glycogen [starch] synthase, liver	0.0215
Q80UG2	Plxna4	1.2501	Plexin-A4	0.0048
Q9Z2H5	Epb41l1	1.2471	Band 4.1-like protein 1	0.0007
Q4G5Y1	Klhdc2	1.2464	Kelch domain-containing protein 2	0.0126
Q99MZ3	Mlxipl	1.2459	Carbohydrate-responsive element-binding protein	0.0169
Q8BMZ5	Tsen34	1.2409	tRNA-splicing endonuclease subunit Sen34	0.0085
Q68FH4	Galk2	1.2403	N-acetylgalactosamine kinase	0.0093
Q61391	Mme	1.2365	Neprilysin	0.0002
Q80ZU0	Arl5a	1.2346	ADP-ribosylation factor-like protein 5A	0.0475
Q0GA42	Cnnm1	1.2343	Metal transporter CNNM1	0.0471
Q8CFA2	Amt	1.2309	Aminomethyltransferase, mitochondrial	0.0056

Q8CH72	Trim32	1.2266	E3 ubiquitin-protein ligase TRIM32	0.0164
Q14DH7	Acss3	1.2259	Acyl-CoA synthetase short-chain family member 3, mitochondrial	0.0130
Q9DBE0	Csad	1.2245	Cysteine sulfinic acid decarboxylase	0.0196
P0DP99	Stmp1	1.2244	Short transmembrane mitochondrial protein 1	0.0252
P00329	Adh1	1.2242	Alcohol dehydrogenase 1	0.0058
P24472	Gsta4	1.2202	Glutathione S-transferase A4	0.0075
Q91ZR5	Catsper1	1.2177	Cation channel sperm-associated protein 1	0.0473
O09117	Sypl1	1.2175	Synaptophysin-like protein 1	0.0407
O54992	Mapkapk5	1.2153	MAP kinase-activated protein kinase 5	0.0268
Q91YA2	Pskh1	1.2141	Serine/threonine-protein kinase H1	0.0231
P56565	S100a1	1.2111	Protein S100-A1	0.0255
P07309	Ttr	1.2099	Transthyretin	0.0004
Q5D1E7	Zc3h12a	1.2084	Endoribonuclease ZC3H12A	0.0015
Q64522	Hist2h2ab	1.2063	Histone H2A type 2-B	0.0473
Q9QXN0	Shroom3	1.2062	Protein Shroom3	0.0034
Q9Z0H4	Celf2	1.2052	CUGBP Elav-like family member 2	0.0067
Q8VCT4	Ces1d	1.2039	Carboxylesterase 1D	0.0195
Q9ES28	Arhgef7	1.2038	Rho guanine nucleotide exchange factor 7	0.0123
Q4ZJM7	Otol1	1.2029	Otolin-1	0.0128
Q91VC9	Ghitm	1.2018	Growth hormone-inducible transmembrane protein	0.0318
Q8R1R3	Stard7	1.2011	StAR-related lipid transfer protein 7, mitochondrial	0.0007

Q91VF2	Hnmt	1.2011	Histamine N-methyltransferase	0.0275

Table S2. Down-regulated hepatic DEPs in *Selenot*-KO mice

Uniprot ID	Gene Name	Fold Change KO/WT	Protein Name	p value
Q8C1B7	Sept11	0.8315	Septin-11	0.0260
Q9CQS8	Sec61b		Protein transport protein Sec61 subunit beta	
Q61704	Itih3	0.8309	Inter-alpha-trypsin inhibitor heavy chain H3	0.0040
Q3TC93	Hs1bp3	0.8292	HCLS1-binding protein 3	0.0201
A6X935	Itih4	0.8290	Inter alpha-trypsin inhibitor, heavy chain 4	0.0280
Q8C754	Vps52	0.8287	Vacuolar protein sorting-associated protein 52 homolog	0.0041
P16294	F9	0.8276	Coagulation factor IX	0.0201
Q8K182	C8a	0.8273	Complement component C8 alpha chain	0.0332
P31001	Des	0.8272	Desmin	0.0163
Q3UJB9	Edc4	0.8263	Enhancer of mRNA-decapping protein 4	0.0243
Q00623	Apoa1	0.8260	Apolipoprotein A-I	0.0207
Q8BH61	F13a1	0.8257	Coagulation factor XIII A chain	0.0147
P51658	Hsd17b2	0.8248	Estradiol 17-beta-dehydrogenase 2	0.0008
Q9JKS5	Habp4	0.8241	Intracellular hyaluronan-binding protein 4	0.0043
Q64378	Fkbp5	0.8233	Peptidyl-prolyl cis-trans isomerase FKBP5	0.0000

Q80X80	C2cd2l	0.8224	Phospholipid transfer protein C2CD2L	0.0008
Q8CH25	Sltm	0.8217	SAFB-like transcription modulator	0.0116
Q8BND5	Qsox1	0.8208	Sulfhydryl oxidase 1	0.0051
Q9D115	Znf706	0.8187	Zinc finger protein 706	0.0047
Q8BM55	Tmem214	0.8179	Transmembrane protein 214	0.0452
Q6ZWY8	Tmsb10	0.8172	Thymosin beta-10	0.0011
Q9JJ00	Plscr1	0.8136	Phospholipid scramblase 1	0.0442
Q8C0L9	Gpcpd1	0.8113	Glycerophosphocholine phosphodiesterase GPCPD1	0.0345
Q9CY24	Tmem179b	0.8111	Transmembrane protein 179B	0.0112
Q8BRH0	Tmtc3	0.8101	Transmembrane and TPR repeat-containing protein 3	0.0085
Q5I012	Slc38a10	0.8095	Putative sodium-coupled neutral amino acid transporter 10	0.0004
Q9D7S7	Rpl22l1	0.8056	60S ribosomal protein L22-like 1	0.0198
Q5NBU8	Xaf1	0.8055	XIAP-associated factor 1	0.0005
Q8BHJ6	Serinc5	0.8055	Serine incorporator 5	0.0162
P15379	Cd44	0.8034	CD44 antigen	0.0306
Q91WP6	Serpina3n	0.8015	Serine protease inhibitor A3N	0.0420
Q8R2E9	Ero1b	0.8015	ERO1-like protein beta	0.0025
P01796		0.8009	Ig heavy chain V-III region A4	0.0430
Q60590	Orm1	0.8006	Alpha-1-acid glycoprotein 1	0.0289
P06339	H2-T23	0.8004	H-2 class I histocompatibility antigen, D-37 alpha chain	0.0271

P62342	Selenot	0.8002	Thioredoxin reductase-like selenoprotein T	0.0275
Q8BWR8	Rhpn2	0.7994	Rhophilin-2	0.0021
Q99KX1	Mlf2	0.7993	Myeloid leukemia factor 2	0.0061
Q3UDW8	Hgsnat	0.7991	Heparan-alpha-glucosaminide N-acetyltransferase	0.0491
Q9ES56	Trappc4	0.7990	Trafficking protein particle complex subunit 4	0.0008
P20152	Vim	0.7969	Vimentin	0.0045
Q61153	Dio1	0.7951	Type I iodothyronine deiodinase	0.0460
Q68FF9	Srd5a1	0.7951	3-oxo-5-alpha-steroid 4-dehydrogenase 1	0.0063
Q80X50	Ubap2l	0.7949	Ubiquitin-associated protein 2-like	0.0022
Q810A3	Ttc9c	0.7941	Tetratricopeptide repeat protein 9C	0.0303
Q9WVB0	Rbpms	0.7908	RNA-binding protein with multiple splicing	0.0322
P31532	Saa4	0.7904	Serum amyloid A-4 protein	0.0159
Q91WE4		0.7888	UPF0729 protein C18orf32 homolog	0.0349
O08692	Ngp	0.7887	Neutrophilic granule protein	0.0182
Q8BLX4	Slc35c1	0.7885	GDP-fucose transporter 1	0.0469
Q62481	Vps72	0.7881	Vacuolar protein sorting-associated protein 72 homolog	0.0326
Q8BHC0	Lyve1	0.7858	Lymphatic vessel endothelial hyaluronic acid receptor 1	0.0180
Q8K0R6	Gltpd2	0.7851	Glycolipid transfer protein domain-containing protein 2	0.0181
O54786	Dffa	0.7848	DNA fragmentation factor subunit alpha	0.0023

Q6NWW9	Fndc3b	0.7839	Fibronectin type III domain-containing protein 3B	0.0437
P23440	Pde6b	0.7805	Rod cGMP-specific 3’,5’-cyclic phosphodiesterase subunit beta	0.0208
P51885	Lum	0.7788	Lumican	0.0336
Q9DCT8	Crip2	0.7785	Cysteine-rich protein 2	0.0420
Q3UIR3	Dtx3l	0.7773	E3 ubiquitin-protein ligase DTX3L	0.0092
Q9JI90	Rnf14	0.7767	E3 ubiquitin-protein ligase RNF14	0.0377
Q9QXM0	Abhd2	0.7760	Monoacylglycerol lipase ABHD2	0.0003
P56873	Sssca1	0.7760	Sjoegren syndrome/scleroderma autoantigen 1 homolog	0.0021
Q9D1M7	Fkbp11	0.7722	Peptidyl-prolyl cis-trans isomerase FKBP11	0.0103
P14873	Map1b	0.7683	Microtubule-associated protein 1B	0.0432
P63254	Crip1	0.7677	Cysteine-rich protein 1	0.0194
Q00898	Serpina1e	0.7626	Alpha-1-antitrypsin 1-5	0.0206
O88904	Hipk1	0.7591	Homeodomain-interacting protein kinase 1	0.0061
Q80YT7	Pde4dip	0.7554	Myomegalin	0.0003
P08207	S100a10	0.7548	Protein S100-A10	0.0287
Q80UY2	Kcmf1	0.7537	E3 ubiquitin-protein ligase KCMF1	0.0158
Q6P8I4	Pcnp	0.7511	PEST proteolytic signal-containing nuclear protein	0.0005
P27005	S100a8	0.7499	Protein S100-A8	0.0214
P11087	Col1a1	0.7482	Collagen alpha-1(I) chain	0.0393
P51437	Camp	0.7419	Cathelicidin antimicrobial peptide	0.0190

Q61646	Hp	0.7401	Haptoglobin	0.0418
Q71KU9	Fgl1	0.7342	Fibrinogen-like protein 1	0.0267
Q01149	Col1a2	0.7313	Collagen alpha-2(I) chain	0.0054
P11588	Mup1	0.7303	Major urinary protein 1	0.0464
A2AAE1	Kiaa1109	0.7279	Uncharacterized protein KIAA1109	0.0259
C0HKD8	Mfap1a	0.7176	Microfibrillar-associated protein 1A	0.0002
Q8BL00	Cdhr3	0.7118	Cadherin-related family member 3	0.0243
Q1ERP8	Cd300lg	0.7068	CMRF35-like molecule 9	0.0384
Q61245	Col11a1	0.7067	Collagen alpha-1(XI) chain	0.0078
Q91Y47	F11	0.7040	Coagulation factor XI	0.0253
Q91WR5	Akr1c21	0.7019	Aldo-keto reductase family 1 member C21	0.0166
P97315	Csrp1	0.6954	Cysteine and glycine-rich protein 1	0.0391
Q9D304	Rnf128	0.6938	E3 ubiquitin-protein ligase RNF128	0.0049
P09602	Hmgn2	0.6922	Non-histone chromosomal protein HMG-17	0.0214
P28654	Dcn	0.6919	Decorin	0.0339
Q9QY24	Zbp1	0.6706	Z-DNA-binding protein 1	0.0435
P14602	Hspb1	0.6493	Heat shock protein beta-1	0.0057
A2ASS6	Ttn	0.6033	Titin	0.0182
Q63836	Selenbp2	0.5763	Selenium-binding protein 2	0.0045
P02802	Mt1	0.5208	Metallothionein-1	0.0019

Table S3. Statistically significant pathways in KEGG pathway analysis of the DEPs in livers of *Selenot*-KO and WT mice.

Pathway Name	P value	P value_ adjusted	Genes|Fold change	Count	First class	Secondary class
**Biosynthesis of unsaturated fatty acids**	0.0213	0.33	Acaa1b|1.43; Scd1|1.415	2	Metabolism	Lipid metabolism
**Glycerophospholipid metabolism**	0.0318	0.358	Cept1|1.315; Pemt|1.261; Gpcpd1|0.811	3	Metabolism	Lipid metabolism
**Fatty acid degradation**	0.0485	0.501	Acaa1b|1.43; Adh1|1.224	2	Metabolism	Lipid metabolism
**Metabolism of xenobiotics by cytochrome P450**	0.0117	0.224	Gsta2|1.273; Adh1|1.224; Gsta4|1.22	3	Metabolism	Xenobiotics biodegradation and metabolism
**Drug metabolism - cytochrome P450**	0.0126	0.224	Gsta2|1.273; Adh1|1.224; Gsta4|1.22	3	Metabolism	Xenobiotics biodegradation and metabolism
**ECM-receptor interaction**	0.0241	0.332	Cd44|0.801; Col1a1|0.742; Col1a2|0.73	3	Environmental Information Processing	Signaling molecules and interaction
**Complement and coagulation cascades**	0.00048	0.0598	F9|0.827; C8a|0.827; F13a1|0.825; Serpina1e|0.759; F11|0.702	5	Organismal Systems	Immune system
**PPAR signaling pathway**	0.00338	0.133	Acaa1b|1.43; Scd1|1.415; Me1|1.272; Apoa1|0.826	4	Organismal Systems	Endocrine system
**Protein digestion and absorption**	0.00429	0.133	Mme|1.236; Col1a1|0.742; Col1a2|0.73; Col11a1|0.704	4	Organismal Systems	Digestive system
**Proteoglycans in cancer**	0.00313	0.133	Itpr3|1.714; Cd44|0.801; Lum|0.779; Col1a1|0.742; Col1a2|0.73; Dcn|0.671	6	Human Diseases	Cancer: overview
**Chemical carcinogenesis**	0.0294	0.358	Gsta2|1.273; Adh1|1.224; Gsta4|1.22	3	Human Diseases	Cancer: overview
**Amoebiasis**	0.00678	0.168	C8a|0.827; Col1a1|0.742; Col1a2|0.73;	4	Human Diseases	Infectious disease: parasitic

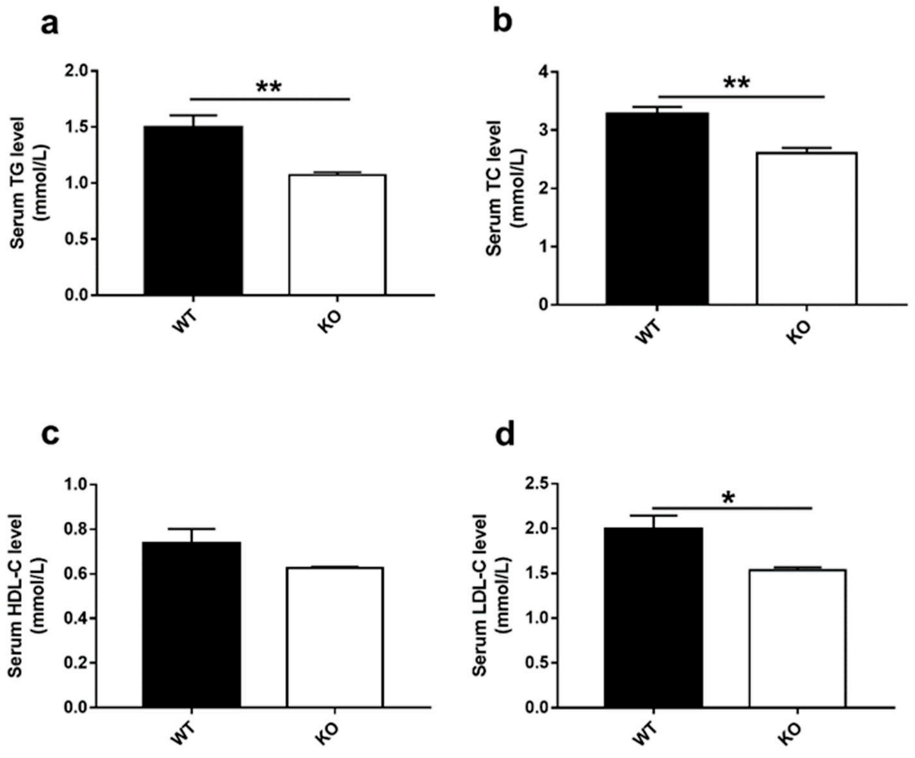

Figure S1. Serum TG, TC, HDL-C, LDL-C levels in male WT and *Selenot*-KO mice. All data are means ± SEM (n = 5-7). * $p < 0.05$, * * $p < 0.01$; ANOVA followed by a mann-whitney nonparametric *U* test.
